# Social Aspects of Suicidal Behavior and Prevention in Early Life: A Review

**DOI:** 10.3390/ijerph9030985

**Published:** 2012-03-19

**Authors:** Maya Amitai, Alan Apter

**Affiliations:** 1 Child and Adolescent Division, Geha Mental Health Center, PetachTikva, Israel; Email: maya47@zahav.net.il; 2 Sackler Faculty of Medicine, Tel Aviv University, Tel Aviv, Israel; 3 Department of Psychiatry, The Feinberg Child Study Center, Schneider Children’s Medical Center of Israel, PetachTikva, Israel

**Keywords:** adolescents, social, cultural, risk factors, suicide, bullying

## Abstract

*Purpose*: The present review summarizes the updated literature on the social aspects of suicidal behavior and prevention in adolescents. *Recent findings*: The predictive role of psychiatric disorders and past history are well recognized in adolescent suicide, but the role of social and cultural factors is less clear. Studies have focused on the importance of ethnicity, gender, family characteristics, and socioeconomic status. More recently, attention has been addressed to broader social risk factors, such as bullying in adolescents, suicide contagion, sexual orientation, and the popular media. Further empirical evidence is needed to advance our understanding of suicidal youth, develop better assessment tools, and formulate effective prevention and treatment programs. *Summary*: Suicidal behavior remains an important clinical problem and major cause of death in youth. Social factors may be at least as important as genetics. Advancing our understanding of underlying cultural and sociological issues in youth suicide will help clinicians achieve more efficient prediction, prevention and treatment.

## 1. Introduction

Knowledge of the risk factors for suicidal behavior in youth has burgeoned during the past 20 years. Converging evidence points topsychiatric or mental disorders as well as a past history of suicidal behavior as the strongest predictors of suicidal behavior and death by suicide [1]. However, the role of social and cultural factors is less clear and remains a topic of theoretical and practical interest. Given that social contextual factors can significantly impact well-being, they might also serve as predictors of suicidal behavior and a basis for formulating preventive measures [2].

The present review focuses on recent developments in our understanding of the social and cultural aspects of youth suicide and suicidal behavior and their implications for prevention. Suicidal behavior is a set of noncontinuous and heterogeneous spectra of behaviors, such that suicidal ideation, suicidal threats, gestures, self-cutting, low lethal suicide attempts, interrupted suicide attempts, near-fatal suicide attempts, and actual suicide may or may not be related to each other, depending on the context in which they are studied [3]. We used the definitions of suicide and suicidal behavior suggested by O’Carroll *et al*. [4]and adopted by the Institute of Medicine, as follows: suicide attempt (SA)—a potentially self-injurious behavior with a non-fatal outcome, for which there is evidence (explicit or implicit) that the person intended at some level to kill himself/herself [4]; non-suicidal self-injury (NSSI)—direct, deliberate destruction of body tissue without lethal intention. [5]; suicidal ideation—any self-reported thoughts of engaging in suicide-related behavior [4]. NSSI and SA are grouped in this review under the term deliberate self-harm (DSH).

## 2. Social Theories of Suicide

According to several major theorists in the field of suicidology, social and cultural variables need to be taken into account in the understanding of suicide. Durkheim, considered the founder of empirical research in sociology and suicidology, hypothesized in his 1897 book *Suicide* that suicide rates vary negatively with the level of social integration (conceptualized as the opposite of anomia, isolation and egoism) of individual groups [6]. He also highlighted the roles of religious integration and varying family circumstances [6]. Since then many biosocial models of self-harmful behavior have incorporated family processes and social support networks [7,8]and support the promotion of social cohesion and identification with societal values in the enhancement of mental health in general and the prevention of suicide in particular. In adolescents, in whom identity is a vital element of well-being [9], this could be accomplished through participation in youth movements, social clubs, sports activities, and national service. Other theories, such as the relational approach of Joiner [10], have implications for the potential benefits of socio-cultural interventions, but these are beyond the scope of this review. 

## 3. Epidemiology: Cultural and Ethnic Issues

Adolescent suicidal behaviors are widespread and produce a significant burden on healthcare systems. In the United States, suicide is the fourth most common cause of death among 10–14-year-olds, and the third most common cause of death among 15–24-year-olds [11]. The epidemiology of adolescent suicide has shown striking changes over the last 100 years, with a steady decline in recent decades. One of the factors suggested to explain this trend is the growing use of antidepressants, especially selective serotonin reuptake inhibitors, in the adolescent population [3].

The prevalence of suicidal behaviors varies significantly across countries, cultures, and racial/ethnic groups worldwide [12]. Even within the same country, there are considerable differences among populations. In the United States, for example, adolescents of Indian/Alaskan descent have the highest rates of fatal suicidal behavior of all ethnic groups, and Latino and Caucasian youth have the highest rates of ideation and DSH [13]. Similarly, extremely high rates of suicide have been recorded for adolescents among theInuit populations in Canada [14] and the Ethiopian population in Israel [15], which share a pattern of a failure of a traditional culture to integrate with modern Western culture.

The large majority of suicides (90.5%) occur among Caucasian Americans. However, the ratefor black adolescent males has been rising significantly and now approximates that of European Americans [12]. Interestingly, only about half of all black adolescent suicide attempters report ever having received a diagnosis of a mental disorder (by accepted criteria); this rate is much lower than rates reported in previous studies of adolescents in general [16]. This finding highlights the importance of moving beyond the study of mental disorders to a broader range of factors to improve our understanding of how suicidal behavior develops.

Another recent epidemiological finding is the variation in the characteristics of youth suicide between Asian and Western countries. In rural China, southern India, Sri Lankaand Singapore, the gender differences for suicide are reversed from those in the West, with young women being at higher risk for suicide than young men; the mode of suicide attempts differs accordingly, consisting mostly of the impulsive use of pesticides [17]. Unlike Western suicidal youth, female attempters in China do not appear to have major mental illnesses [18]. These data have important theoretical and preventive implications. 

## 4. Risk Factors for Suicidal Behavior

Major established risk factors for suicide in youth include a previous suicide attempt, availability of lethal means, and family discord [19]. However, most of the studies focused on Caucasian youth, and less is known about the suicidal behavior of ethnic minorities. This section covers some of the important social risk factors underlying adolescent suicidal behavior.

### 4.1. Gender

In Western countries, the rates of suicide across ethnicities are higher in adolescent boys than adolescent girls (ratio of 5:1), whereas the rates of suicidal ideation and attempted suicide are higher in girls (ratio of 3:1) [20]. Explanations for the higher suicide rate in boys include higher suicidal intent, use of more violent methods, higher prevalence of antisocial disorder and substance abuse, and greater vulnerability to stressors, such as legal difficulties, financial problems, and interpersonal loss [21]. Boys may also have more difficulties in asking for help and communicating their distress [22]. The gender gap inDSH is most pronounced among youths of Caucasian American descent and least pronounced among American Indians [23]. The gender gap in suicide mortality has been widening in recent decades, especially in some ethnic minority groups in the United States, mostly because of the increase in suicide among ethnic minority boys accompanied by stable suicide mortality rates among girls of all ethnic groups.

A key issue in adolescent suicidal behavior is the different impact of certain risk factors by gender. Some risk factors lead to different suicidal behaviors (fatal/nonfatal) in boys and girls, and others are associated with suicidal behavior specifically in girls but not boys or vice versa. For example, depression appears to be a better predictor of suicidal behavior in European American girls than boys, whereas alcohol abuse, substance abuse, and conduct disorders appear to be stronger correlates of suicidal behavior in European American boys than girls. In the United States, sexual abuse is increasingly being recognized as a factor in girls’ DSH. Conflict with parents seems to create a unique vulnerability of girls to DSH [23]. Others found that social isolation from peers and intransitive friendships significantly increase the odds of suicidal ideation in girls, and being part of a tightly networked school community (high relative density of friendship ties) is protective against suicide attempts in boys. Thus, social network variables are relevant to suicidality in different ways inboys and girls [24].

Accordingly, there may be preventive methods that are more suitable for one gender than the other. As mentioned, in Western cultures, adolescent males often find it difficult to seek help owing to social norms [25]. Therefore, encouraging adolescent boys to communicate distress before it is too late should be a cornerstone of school and youth suicide preventive programs. This could be especially useful for young military conscripts. By contrast, girls should be encouraged to adopt more constructive coping mechanisms rather than self-injury as a means of solving interpersonal problems. Recent developments in feminist psychology, such as the practices introduced by Carol Gilligan, may be very helpful in this regard [26]. Gilligan offers gender-based strategies for preventing psychological distress and youth violence. According to Gilligan, girls tend to suicidal behavior as a language that commands attention and respect and as an expression of a desire for relationship, while boys turn to violence as an alternativeto feeling helpless and powerless. Thus, shifting the interpretation of the suicidal behavior to the relational communication of the violent intention might enable adolescent girls to verbally express their psychological distress. Moreover, strengthening healthy resistance and couragein young children (boys and girls) will prevent violence and enable these young adolescents to say what they feeland to know how to stay in a relationship with others instead of turning to suicidal behaviors.

### 4.2. Family Factors

Research has pointed to the importance of the family environment as a predictor of suicidal behavior among adolescents. The relevant family-related risk factors are parental psychopathology, family history of suicidal behavior, family discord, loss of a parent to death or divorce, poor quality of the parent-child relationship, and maltreatment [16]. There is strong and convergent evidence that suicidal behavior is familial and, perhaps, genetic, and that the liability to suicidal behavior is transmitted in families independently of psychiatric disorder [27]. Nevertheless, there may also be environmental routes of transmission, such as imitation and intergenerational family adversity [28]. Therefore, prevention programs should be designed for early identification and treatment of potentially suicidal adolescents from dysfunctional families. Mental health professionals should be encouraged to try to improve functioning within the family of suicidal youth.

### 4.3. Physical and Sexual Abuse

Empirical studies overwhelmingly point to an association between childhood abuse/neglect and suicidality for both boys and girls and within different ethnic/racial groups [29]. Exposure tophysical and, especially, sexual abuse in childhood leads to a significant increase in poor mental health outcomes, including suicidal ideation and behavior, experienced at ages 16 to 25 [30]. The risk is increased if the child is sexually abused by an immediate family member or the sexual abuse is repeated over time [31]. The greater the severity of the abuse, the higher the risk of suicide attempts [31]. Interestingly, Garnefski and Arends [32] found that sexually abused boys were at greater risk of suicide attempts than sexually abused girls, although both groups were at higher risk than non-abused boys and girls [32]. Thus, all abused children and adolescents should be carefully evaluated for suicidal thoughts and behaviors, and health professionals who work with them should be trained in adolescent suicidal therapy. Several interventions have been investigated as strategies to prevent suicide in abused children, including family preservation or unification models, broad ecologically based intervention models and prevention models.One of the largest projects examined mental health interventions for children who were victims of intrafamilial physical or sexual abuse. Trauma-focused cognitive-behavioral therapy was proven effective in reducing psychological distress in these children [33]. Moreover, it seems that better education regarding reporting suspected abuse and making it easier for children to seek help if they are being abused may also be important measures.

### 4.4. Change of Residence and Socioeconomic Class

Qin *et al*. [34]reported that children who frequently moved were more likely to make suicide attempts during adolescence. There was a dose-response relationship between number of moves and risk of attempted suicide. However, other studies found that residential mobility was associated with suicide attempts in adolescent females but not males, suggesting an important gender difference [35]. More empirical research is needed in order to address this difference. Another factor is social class. Some studies show that adolescents who engage in DSH behaviors tend to be from lower socioeconomic strata [36,37], while other studies found no such association. Additionally, low levels of parental education are associated with higher adolescent suicidal risk [23].

### 4.5. Sexual Orientation

Youth who report same-sex sexual orientation are at greater risk than their peers to attempt suicide, and this risk persists even after controlling for other suicide risk factors [38]. According to a recent study, gay, lesbian or bisexual adolescents who experience family rejection or a negative family reaction at their “coming out” have an eightfold greater likelihood of attempting suicide than adolescents who experience no or minimal family rejection [39]. These findings indicate that providing the gay/lesbian adolescent community with help in resolving their identity issues is an important part of suicide prevention. Moreover, addressing the societal rejection issue seems to be an important measure in this regard.

### 4.6. Alcohol and Drugs

Alcohol abuse is known to be associated with an increased risk of suicidal behavior and suicide death among adolescents. A recent study reported that the link between heavy episodic drinking (HED) and suicide attempts is maintained even after controlling for depression [40]. The association was strongest in the under 13-year age group and decreased with increasing age. These findings suggest that early HED may be a marker for some other factor (e.g., poor behavioral inhibition, poor decision making, cognitive precociousness) causally related to suicide attempts [12]. Restricting alcohol sales to adolescents has already been shown to be an effective suicide-prevention measure [41,42].

### 4.7. Bullying

Klomek *et al.* [43]showed that bullying and victimization during childhood increase the odds of a subsequent suicide attempt [43]. However, Brent *et al*. [20] found that in boys, bullying, but not victimization, was associated with suicide, but the association was not causal; rather, both bullying and suicide were both consequences of conduct disorder, a known risk factor for suicidal behavior. By contrast, in girls, victimization, but not bullying, was associated with suicide attempts, even after adjusting for conduct disorder and depression [21]. Others reported that boys who were both bullies and victims of bullying had a higher likelihood of suicidal behavior than boys who were only victims [43]. In girls, victims of bullying were more likely to exhibit suicidal behaviors than those who were neither bullies nor victims [43]. Today, many youth are subject to cyber-bullyingthrough Email, cell phone texting, and internet social sites, perpetrated by other adolescents or even adults [41]. These findings call for strenuous efforts by school authorities to prevent bullying and the formulation of interventions to minimize its deleterious effects, particularly regarding cyber-bullying.

### 4.8. Suicide Contagion

Social learning may be an important factor in both familial and nonfamilial transmission of suicidal behaviors. The concept of suicide contagion is based on the infective disease model and assumes that a suicidal behavior by one person may facilitate the occurrence of subsequent, similar behaviors by others [44]. The process is implemented via imitation. Theories of imitation have been postulated to explain clustering of suicides and DSHbehaviors. Studies conducted primarily in adolescents revealed that up to 5% of all suicides may be the result of suicide clustering and that exposure to DSH behaviors in family and friends was predictive of DSH and suicide ideation [44]. A large body of research in the last 10 years clearly demonstrated that extensive newspaper and television coverage of suicide is associated with a significant increase in the rate of suicide [45]. The magnitude of the increase is proportional to the amount, duration, and prominence of the media coverage. This phenomenon is termed the “Werther effect” after Goethe’s novel, *The Sorrows of Young Werther* (1774), which was assumed to have triggered an increase in suicides after its release. As a result, the book was banned in many European countries. Today, the increasing popularity of the Internet as a source of information has raised concerns about the danger of sites that promote suicide and sites set up by strangers who form suicide pacts [46]. Further empirical research is needed to clarify their effects. By the same token, however, the media may also serve as an effective means for preventing suicide contagion. More efforts should be directed at presenting stories of suicide, especially by persons admired by youth, in a different light. One successful example is the media’s treatment of the suicide of the guitarist and singer, Kurt Cobain [47]. The lack of an apparent copycat effect following Cobain’s death is hypothesized to be due to various aspects of the media coverage and the intense activity of the crisis center and community outreach interventions in Seattle that occurred following Cobain’s suicide.

## 5. Preventive Measures

Despite the heavy burden that adolescent suicidal behaviour imposes on individuals and communities, little is known about effective preventive measures. Of the few studies that have investigated such interventions, most were targeted at adults and reported only moderate effectiveness [48]. More attention is now being addressed at school-based prevention programs, which hold particular promise because teachers and other school staff can serve as “gatekeepers” or “gateway providers,” spotting students who seem to be in turmoil and referring them to mental health services [49]. This approach is noteworthy because the latest research suggests that most suicidal youth do not receive mental health careor even tell an adult about their suicidal thoughts or behaviors [49]. Furthermore, there are many innovative prevention efforts directed at ethnic minorities in which suicidal behavior has become epidemic, such as native Indians of Arizona [50] and the Inuit in Canada [51]. They focus on restoring ethnic pride and cultural values using a “bottom-up” approach, starting with intensive consultation with the local community. Further research is needed to understand the phenomenology of suicidal behaviors among ethnic minority populations [13], including the presentations of suicidal behavior, meanings of suicidal behavior in different cultures, risk factors particular to these groups and their correlates, applicability of known risk factors in other populations, such as depression, and preventive mechanisms.

## 6. Conclusions

Suicide remains an important clinical problem and a major cause of death in young people. The role of social factors in suicidal behavior is long established, and there is compelling evidence that they may be at least as prominent as genetic factors. Knowledge on effective interventions in adolescents who attempt suicide, cause DSH, or have suicidal ideations is still extremely limited, and methodologically rigorous trials are required. Advancing our understanding of cultural and sociological issues underlying suicidal behavior is critical to prevention in diverse societies. In addition, the identification of more specific risk factors will help clinicians predict suicidality and target preventive treatment. In this context, the concepts propagated by the classical theories of Durkheim and Erickson are still highly relevant today.

## References

[1] Brent D.A., Perper J.A., Moritz G., Allman C., Friend A., Roth C., Schweers J., Balach L., Baugher M. (1993). Psychiatric risk factors for adolescent suicide: A case-control study.. J. Am. Acad. Child. Adolesc. Psychiatry.

[2] King C.A., Merchant C.R. (2008). Social and interpersonal factors relating to adolescent suicidality: A review of the literature.. Arch. Suicide Res..

[3] Bursztein C., Apter A. (2009). Adolescent suicide.. Curr. Opin. Psychiatry.

[4] O’Carroll P.W., Berman A.L., Maris R.W., Moscicki E.K., Tanney B.L., Silverman M.M. (1996). Beyond the Tower of Babel: A nomenclature for suicidology.. Suicide Life Threat. Behav..

[5] Nock M.K. (2009). Why do people hurt themselves? New insights into the nature and functions of self-injury.. Curr. Dir. Psychol. Sci..

[6] Durkheim E. (1897). Suicide.

[7] Linehan M.M. (1993). Cognitive-Behavioral Treatment of Borderline Personality Disorder.

[8] Cohen S., Wills T.A. (1985). Stress, social support, and the buffering hypothesis.. Psychol. Bull..

[9] Erikson E. (1959). Identity and the life cycle: Selected papers.. Psychol. Issues.

[10] Joiner T. (2006). Why People Die by Suicide.

[11] Anderson R.N., Smith B.L. (2003). Deaths: Leading causes for 2001.. Natl.Vital Stat. Rep..

[12] Nock M.K. (2009). Suicidal behavior among adolescents: Correlates, confounds, and (the search for) causal mechanisms. J. Am. Acad. Child. Adolesc. Psychiatry.

[13] Joe S., Canetto S.S., Romer D. (2008). Advancing prevention research on the role of culture in suicide prevention. Suicide Life Threat. Behav..

[14] Wexler L., Hill R., Bertone-Johnson E., Fenaught A. (2008). Correlates of Alaska Native fatal and nonfatal suicidal behaviors 1990–2001.. Suicide Life Threat. Behav..

[15] Shoval G., Schoen G., Vardi N., Zalsman G. (2007). Suicide in Ethiopian immigrants in Israel: A case for study of the genetic-environmental relation in suicide.. Arch. Suicide Res..

[16] Bridge J.A., Goldstein T.R., Brent D.A. (2006). Adolescent suicide and suicidal behavior.. J. Child. Psychol. Psychiatry.

[17] Law S., Liu P. (2008). Suicide in China: Unique demographic patterns and relationship to depressive disorder.. Curr. Psychiatry Rep..

[18] Phillips M.R., Yang G., Zhang Y., Wang L., Ji H., Zhou M. (2002). Risk factors for suicide in China: A National case-control psychological autopsy study.. Lancet.

[19] Cash S.J., Bridge J.A. (2009). Epidemiology of youth suicide and suicidal behavior.. Curr. Opin. Pediatr..

[20] Eaton D.K., Kann L., Jinchen S., Shanklin S., Ross J., Hawkins J., Harris W.A., Lowry R., McManus T., Chyen D. (2008). Youth risk behavior surveillance—United States, 2007.. MMWR Surveill. Summ..

[21] Brent D.A., Baugher M., Bridge J., Chen T., Chiappetta L. (1999). Age- and sex-related risk factors for adolescent suicide.. J. Am. Acad. Child Adolesc. Psychiatry.

[22] Levi Y., Horesh N., Fischel T., Treves I., Or E., Apter A. (2008). Mental pain and its communication in medically serious suicide attempts: An “impossible situation”.. J. Affect. Disord..

[23] Canetto S.S. (1997). Meanings of gender and suicidal behavior during adolescence.. Suicide Life Threat. Behav..

[24] Bearman P.S., Moody J. (2004). Suicide and friendships among American adolescents.. Am. J. Public Health.

[25] Apter A., Bleich A., King R.A., Kron S., Fluch A., Kotler M., Cohen D.J. (1993). Death without warning? A clinical postmortem study of suicide in 43 Israeli Adolescent males.. Arch. Gen. Psychiatry.

[26] Gilligan C. (2004). Strengthening healthy resistance and courage in children: A gender-based strategy for preventing youth violence.. Ann. N. Y. Acad. Sci..

[27] Brent D.A., Mann J.J. (2005). Family genetic studies, suicide, and suicidal behavior.. Am. J. Med.Genet. C.

[28] Brent D.A., Melhem N. (2008). Familial transmission of suicidal behavior.. Psychiatr. Clin. North Am..

[29] Brodsky B.S., Mann J.J., Stanley B., Tin A., Oquendo M., Birmaher B., Greenhill L., Kolko D., Zelazny J., Burke A.K. (2008). Familial transmission of suicidal behavior: Factors mediating the relationship between childhood abuse and offspring suicide attempts.. J. Clin. Psychiatry.

[30] Fergusson D.M., Boden J.M., Horwood L.J. (2008). Exposure to childhood sexual and physical abuse and adjustment in early adulthood.. Child Abuse Negl..

[31] Brezo J., Paris J., Vitaro F., Hebert M., Tremblay R.E., Turecki G. (2008). Predicting suicide attempts in young adults with histories of childhood abuse.. Br. J. Psychiatry.

[32] Garnefski N., Arends E. (1998). Sexual abuse and adolescent maladjustment: Differences between male and female victims.. J. Adolesc..

[33] chaffin M., Friedrich B. (2004). Evidence-based treatments in child abuse and neglect.. Child.Youth Serv.Rev..

[34] Qin P., Mortensen P.B., Pedersen C.B. (2009). Frequent change of residence and risk of attempted and completed suicide among children and adolescents.. Arch. Gen. Psychiatry.

[35] Haynie D.L., South S.J., Bose S. (2006). Residential mobility and attempted suicide among adolescents: An individual-level analysis.. Sociol. Q..

[36] Ayton A., Rasool H., Cottrell D. (2003). Deliberate self-harm in children and adolescents: Association with social deprivation.. Eur. Child. Adolesc. Psychiatry.

[37] Kokkevi A., Rotsika V., Arapaki A., Richardson C. (2012). Adolescents’ self-reported suicide attempts, self-harm thoughts and their correlates across 17 European countries. J. Child. Psychol. Psychiatry.

[38] Russell S.T., Joyner K. (2001). Adolescent sexual orientation and suicide risk: Evidence from a National study.. Am. J. Public Health.

[39] Ryan C., Huebner D., Diaz R.M., Sanchez J. (2009). Family rejection as a predictor of negative health outcomes in white and Latino lesbian, gay, and bisexual young adults.. Pediatrics.

[40] Aseltine R.H., Schilling E.A., James A., Glanovsky J.L., Jacobs D. (2009). Age variability in the association between heavy episodic drinking and adolescent suicide attempts: Findings from a large-scale, school-based screening program.. J. Am. Acad. Child. Adolesc. Psychiatry.

[41] Mann J.J., Apter A., Bertolote J., Beautrais A., Currier D., Haas A., Hegerl U., Lonnqvist J., Malone K., Marusic A. (2005). Suicide prevention strategies: A systematic review.. J. Am. Med. Assoc..

[42] Nicholson M., Hoye R. (2009). Reducing adolescents’ exposure to alcohol advertising and promotion during televised sports.. J. Am. Med. Assoc..

[43] Klomek A.B., Sourander A., Niemela S., Kumpulainen K., Piha J., Tamminen T., Almqvist F., Gould M.S. (2009). Childhood bullying behaviors as a risk for suicide attempts and completed suicides: A population-based birth cohort study.. J. Am. Acad. Child. Adolesc. Psychiatry.

[44] De Leo D., Heller T. (2008). Social modeling in the transmission of suicidality.. Crisis.

[45] Gould M.S. (2001). Suicide and the media.. Ann. N. Y. Acad. Sci..

[46] Biddle L., Donovan J., Hawton K., Kapur N., Gunnell D. (2008). Suicide and the Internet.. BMJ.

[47] Jobes D.A., Berman A.L., O’Carroll P.W., Eastgard S., Knickmeyer S. (1996). The Kurt Cobain suicide crisis: Perspectives from research, public health, and the news media. Suicide Life Threat. Behav..

[48] Wasserman D., Carli V., Wasserman C., Apter A., Balazs J., Bobes J., Bracale R., Brummer R., Bursztein-Lipsicas C., Corcoran P. (2010). Saving and empowering young lives in Europe (SEYLE): A randomized controlled trial.. BMC Public Health.

[49] Freedenthal S. (2010). Adolescent help-seeking and the Yellow Ribbon Suicide Prevention Program: An evaluation.. Suicide Life Threat. Behav..

[50] Mullany B., Barlow A., Goklish N., Larzelere-Hinton F., Cwik W., Craig M., Walkup J.T. (2009). Toward understanding suicide among youths: Results from the White Mountain Apache tribally mandated suicide surveillance system, 2001–2006. Am. J. Public Health.

[51] Kral M., Wiebe P.K., Nisbet K., Dallas C., Okalik L., Enuaraq N., Cinotta J. (2009). Canadian Inuit community engagement in suicide prevention.. Int. J.Circumpolar Health.

